# Impact of COVID-19 on quality checks of solid tumor molecular diagnostic testing-A surveillance by EQAS provider in India

**DOI:** 10.1371/journal.pone.0274089

**Published:** 2022-09-22

**Authors:** Omshree Shetty, Tanuja Shet, Ramya Iyer, Prachi Gogte, Mamta Gurav, Pradnya Joshi, Nupur Karnik, Trupti Pai, Sridhar Epari, Sangeeta Desai

**Affiliations:** Department of Pathology, Molecular Pathology Laboratory, Tata Memorial Hospital, Homi Bhabha National Institute, Mumbai, India; Food and Drug Administration, UNITED STATES

## Abstract

**Background:**

Molecular tests in solid tumours for targeted therapies call for the need to ensure precision testing. To accomplish this participation in the External Quality Assessment Program (EQAS) is required. This evaluates the consistency of diagnostic testing procedures and offers guidance for improving quality. Outbreak of COVID-19 pandemic led to worldwide lockdown and disruption of healthcare services including participation in EQAS.The present study describes the extended scope of EQAS offered byMPQAP (Molecular Pathology Quality Assurance Program), the first proficiency test provider for solid tumor diagnostics in India. The study surveys the preparedness of molecular testing laboratories in routine diagnostics and participation for quality assessment scheme.

**Methods:**

A documented guideline for measures and precautions to be carried by testing laboratories in performing routine diagnostic tests during the lockdown period were charted and distributed to all MPQAP participant centres. A survey was conducted for MPQAP participants to check whether laboratories were involved in COVID-19 testing and to evaluate the impact of lockdown on the operations of diagnostics procedures. From the acquired response of the survey, 2 cycles out of initially proposed 11 cycles were executed with transformed approach using digital tools and image interpretation modules.

**Findings:**

Out of 25 solid tumour testing laboratories registered as participants, 15 consented to participate in survey. The summary of survey conveyed the impact of COVID-19onroutine operations of diagnostics tests such as shortcomings in inventory and human resource management. Thirteen participants showed active willingness and consented to participate in EQAS test scheme.

**Interpretations:**

The survey findings and assessment of EQAS cycles endorsed the quality testing procedures carried by participating laboratories throughout the lockdown. It highlighted the utility of EQAS participation during pandemic along with emphasis on safety measures for continual improvement in quality of diagnostic services.

## Introduction

Molecular diagnostic tests provide the requisite guidance for target-based therapy in solid tumours, which plays an integral part of diagnostic arsenal for improved management of cancer and keeping up with the current standards [1]. The priority is given to the quality of diagnostic information to be conveyed to the clinician to enable appropriate decision making [2]. Detection of differential mutations at genome level using advanced gene panels and testing platforms has led to generation of prompt test results. However, the sensitivity and reliability of these molecular results can be limited by the myriad factors such as tumor heterogeneity, representative tumor area, tissue adequacy, tissue processing, technical performance and analytical interpretation [3]. Minimizing these errors is improved by continuous participation in EQAS as demonstrated by several studies, thus emphasising on maintaining high standards of quality testing by laboratories [4, 5].

Numerous proficiency testing (PT) providers and EQAS bodies have been set up across the globe for oncology diagnostics, out of which limited organizations probe into solid tumor based testing. EQAS program for oncology in India has been instigated by National Cancer Grid (NCG) in form of ‘MPQAP’ (Molecular Pathology Quality Assurance Program). MPQAP EQAS is the first initiative in the country providing PT schemes for solid tumor molecular diagnostics. It follows Method based Proficiency testing (MBPT) approach that assess the skills of participant laboratories in various aspects of testing. These include performance in technical skills, diagnosis and interpretative skills with compliance to the standard recommendations. MPQAP has been continuously rendering these services across the country since 2019 for various types of molecular diagnostics tests based on methods namely FISH (*Fluorescent in-situ hybridization*), Sanger Sequencing, PCR and Real time PCR (Table 1).

**Table 1:** Test schemes covered under MPQAP EQAS program for solid tumors.

Assay Modules	Test			
**1.FISH**	*HER2/neu*	*MYCN*	*ALK-1*	*ROS1*
	*MET*	*EWSR1*	*1p19q*	*EGFR*
**2.PCR**	Sarcoma Translocations			
**3.RT-PCR**	*EGFR*			
**4. Gene Sequencing**	*BRAF*	*RAS*	*KIT*	*PDGFRA*
	*IDH1/IDH2*			
**5.Clonality Assay**	B Cell & T Cell gene arrangement			

The outbreak of Severe Acute Respiratory Syndrome Coronavirus-2 (SARS-CoV-2) also known as COVID-19,declared as pandemic by the World Health Organization (WHO)professed an imposing threat to health care providers and patients [6]. With limited information and uncertainty of available therapeutic options to contain the spread of transmission the novel virus posed a great threat and panic. This led to unprecedented demand for healthcare resources in clinical testing laboratories. Due to rapid spread of the virus through social contact, emphasis had been given only to single opportunity minimal invasive and sampling procedures for onco-molecular tests to avoid the hazards of transmission. While foremost priority was given to COVID-19 detection and then providing treatment to subsequent diagnosis of any disease, it became an imperative requirement for testing laboratories to function in such a way where quality of conventional molecular diagnostic tests is not hampered under drift of the pandemic restrictions.

In solid tumor molecular diagnostics, the journey of the tissue encompasses three stages of testing-pre-analytical (type of specimen, method of fixation, type of fixatives, tumor content, adequacy, representative tumor tissue), analytical (quality and quantity of the nucleic acids, type of the assay, limit of detection, assay sensitivity) and post-analytical (raw data quality, analysis, interpretation with nomenclature of the genetic alterations and tier-based classification). Such schematic plan involves interdisciplinary team of pathologist, laboratory scientists and scientific assistants to provide an integrated diagnosis [3]. This intricate network of diagnostics was disoriented due to changes in institutional staffing norms to accommodate social distancing; cross-sectoral skill adjustments leading to additional shared responsibilities, travel restrictions due to the lockdown and reduced workforce due to staff in quarantine/isolation. Such circumstances challenge the ongoing confidence of testing laboratories in their performance.

Additionally, as per international bodies such as ISO 15189/CLIA (Clinical Laboratory Improvement Amendments), it is an obligate need for oncology diagnostic laboratories to involve in PT schemes in order to ensure that Good Laboratory Practices(GLP) and competency in testing are maintained [7]. Although, the internal quality control systems (IQMS) setup by individual laboratories are robust, they are limited to assess the performance of testing laboratory with respect to analytical procedure, reagents, equipment, operator and interpretations. Retrospective schemes of EQAS programs offers competency evaluation of participant laboratories in testing of samples that simulate routine diagnostic setting [5].

Prior to pandemic, MPQAP had been instrumental in giving suggestions for improvement of participant laboratories’ efficacy in diagnostic testing and reporting of results through confidential assessment reports (CAR). This role was impeded due to ongoing COVID-19 crisis.

Recognizing the health emergency, international accreditation bodies and PT providers such as CLIA and College of American Pathologists (CAP)had adopted lenient approaches for PT of participating laboratories with the following points [8]:

- Exemption from being penalized for delay in submission of results, suspension or cancellation of participation
- Non-withdrawal from participation, if involved in COVID-19 testing or temporarily suspension of diagnostic tests due to shortage of manpower and reagents
- Exemption from evaluation in situations of discontinuation of diagnostic tests
- Extended time scales for delayed receipt of testing items
- Exemption from participation, if test material is received in unsuitable conditions or unfit for testing

Likewise, guidelines were laid down by WHO and Centre for Disease Control (CDC) for safe operation of laboratories [9–11]. Also, the American Society of Clinical Oncology(ASCO) offered short term practices to follow while providing cancer care during pandemic [12].

With reference to such initiatives at international level, it was deemed essential to have documented measures and strategies to be followed in molecular oncology testing compassing brief points to be employed in the country to resume elective procedures [6, 13].

Since molecular diagnostic work in solid tumours mainly constitutes Formalin Fixed Paraffin Embedded (FFPE) blocks and blood samples; it was imperative to introduce proper risk assessment for procedures such as isolation of nucleic acids, PCR, sequencing and staining. Also, molecular pathology work being non-propagative in nature, it entailed the need to carry all procedures in level II Biosafety Cabinet (BSL-2) or facilities equivalent to it [14].

The responsibility of PT providers expands in such situations where it plays a valuable service of monitoring performance and recommending guidelines for quality sustenance [15]. In contrast to the usual notion that participation in EQAS only helps in identification of diagnostic errors, minimizing it and aiding improvisation, the scope of PT providers also extends in proposing the use of alternate assays or procedures (AAP) for testing to circumvent the crisis of consumables and kits. Thus reinforcing the fact that participation in EQAS not only helps in maintaining quality assurance for the diagnostics but also acts as an essential factor for laboratory credibility system [16].

The present study describes the extended role played by the MPQAP as EQAS provider in assessing the preparedness of testing laboratories during the pandemic and fortifying the importance of quality sustenance in testing procedures even amidst the crisis.

## Materials and methods

The MPQAP PT program was approved by the Institutional Ethics committee (IEC) of Tata Memorial Hospital (TMH) and was initiated in the year 2019. This program has been offering EQAS schemes to 25 participant testing laboratories in India registered under MPQAP. The details of the registration procedure are available on the NCG-MPQAP webpage on the TMH website [14].The test covered under the MPQAP program is provided in Table 1.

### Release of interim guidelines for MPQAP participants

At the inception of pandemic, a documented guideline including interim actions to be undertaken related to laboratory biosafety in handling and processing clinical samples/infectious agents was outlined by NCG-MPQAP coordinators. This guideline was offered to all participants via email and was also uploaded on NCG-MPQAP website [14] (S1 File).

The interim guidelines to be followed in molecular diagnostic laboratory for routine procedures concentrated on patient and healthcare worker safety is summarized in Table 2. The guidelines briefed upon the following leads:

- Staff Safety
- Use of PPE
- Collection & handling of specimens
- Routine diagnostic procedures
- Spill Clean up
- Waste Management

**Table 2:** Summary of precautionary measures to be taken for dealing with patients and diagnostic samples.

Patient and HCW Safety	Pre-Analytical	Analytical	Post- Analytical
Active screening and segregation of symptomatic, asymptomatic and immunosuppressive therapy patients	Use of appropriate N95/ FFP2 masks and PPE for dealing with suspected patient samples	Follow Good Laboratory Practices	Discard the specimens in color coded bags as per BMWM rules
Active monitoring of HCWs for symptoms and exposure	Ensure transport of samples in leak-proof biohazard specimen bags -triple packaging	Use disposable, powder free gloves and avoid frequent reuse or disinfection	Use of 1% Sodium hypochlorite for decontamination and spillage
Dedicated kiosks for sample collection, report despatch and billing with 6-foot distancing markings	Process initial steps of testing in BSC Level II	Disinfect the work area with 70% alcohol; Ensure separate working areas to avoid cross contamination	Discard COVID-19 Waste in proper segregated bags and label them as “COVID-19 Waste”
Strict Social distancing norms at cafeteria, workstations and during assemblies		Use PPE for specimen processing and work in BSC Level II for aerosol, spillage associated procedures	

BSC Level II-Biosafety Cabinet; BMWM-Bio-medical Waste Management; PPE-Personal Protective Equipment

### Assessing the preparedness of the participant laboratories through survey

A survey was designed to ascertain the current testing status of participant laboratories associated with MPQAP EQAS. The objective of the survey was to identify which testing laboratories were involved in COVID-19 testing apart from routine molecular diagnostics and whether adequate facilities were available within them to carry all these tests efficiently. The survey was carried out using Google survey forms with 15 questions taking approximately 5 minutes to complete. Participants who consented to take up the survey were given a duration of 30 days to fill the survey form. The survey responses were collected and collated in June 2020(S2 File).

The survey questions addressed the following points:

1. Whether the testing laboratories are involved in COVID-19 testing
2. Whether adequate supply of consumables is available
3. Impact on diagnostic test requisitions
4. Shortage of reagents, kits and consumables
5. Impact on manpower, revenue and remuneration
6. Impact on Turnaround time (TAT) for reporting
7. Quality measures employed
8. Ready/willingness to participate in EQAS cycle

### Initiation of the PT cycle for the participant laboratories

Prior to inception of pandemic, the calendar schedule of EQAS cycles for the year 2020–2021 had been prepared and was uploaded on MPQAP website (S1 Table). The schedule was to release PT materials on monthly basis for each test covered under various techniques such as FISH, Sanger Sequencing, PCR and Real time PCR. However due to lockdown restrictions, the dispatch schedule was affected.

After assessing the status quo of testing laboratories from the survey responses and the willingness to participate in EQAS cycle, the release of test cycles was planned. To ensure COVID-19 safety and social distancing norms are followed and workflow of pre to post analytics is not hampered, a hybrid design for EQAS cycles was implemented. As per the proposed calendar schedule of MPQAP EQAS cycles of the annual year, two cycles were executed in sequential manner after obtaining consent from participant laboratories.

### Execution of EQAS cycles with improvisation

The first EQAS cycle for FISH module was executed for *Her2/neu* gene amplification test. The consent and confirmation for participation was taken via email and thirteen participants consented to take part in the EQAS cycle. When the inter-state shipment protocols were eased across the country after four months of total lockdown (i.e. from mid-March 2020 to mid-July 2020), the package of test materials were dispatched to the participant centres.

In view of the ongoing pandemic, the design of the test scheme was modified to meet the demands of remote reporting, cloud-based data analysis.

Digital software tools and digital slide scanners were used for obtaining Haematoxylin and eosin (H&E) slide images. These were utilized with the purpose of sending scanned H&E images of the tissue samples to consultant pathologists for remote reporting on virtual platforms to assess the representative tumor content and histology.

H&E stained slides were scanned using whole slide imaging (WSI)system, VENTANA DP200 whole-slide scanner (Hemel Hempstead, UK). Unstained slides for performing FISH test and virtual slide images by WSI of H&E slides of the tumour tissues, were provided as test materials for the cycle. Sending the hard copy of testing results was kept optional and provision was made to submit test results via a dedicated online MPQAP portal system.

### Digital EQAS cycle

With reliability of cloud-based analysis tools ensuring social distancing, the theme of remote reporting was utilised and digital EQAS scheme was further implemented for the next cycle of *RAS* and *BRAF* mutation detection. Four participants consented via email to participate in the cycle.

To assess the interpretative skills of participant laboratory apart from technical quality assessment, Image Interpretation Skill EQAS scheme was executed where digital images were sent as test materials. Participant centres which employ only sequencing methods for detection of *RAS and BRAF* mutation participated in the scheme.

Five digitised images of sequencing data generated using Chromas Lite Sequencing Analysis Software (Version 2.6.1) for each *BRAF* and *RAS* samples were sent to participants via Google forms in their registered email ids (S3 File).

Apart from the digital images, FFPE tissue sections were also provided as test materials for assessing the technical performance of participants.

## Results

### Survey responses of the participant laboratories

#### 1. Centers involved in COVID-19 testing

**Fig 1:**
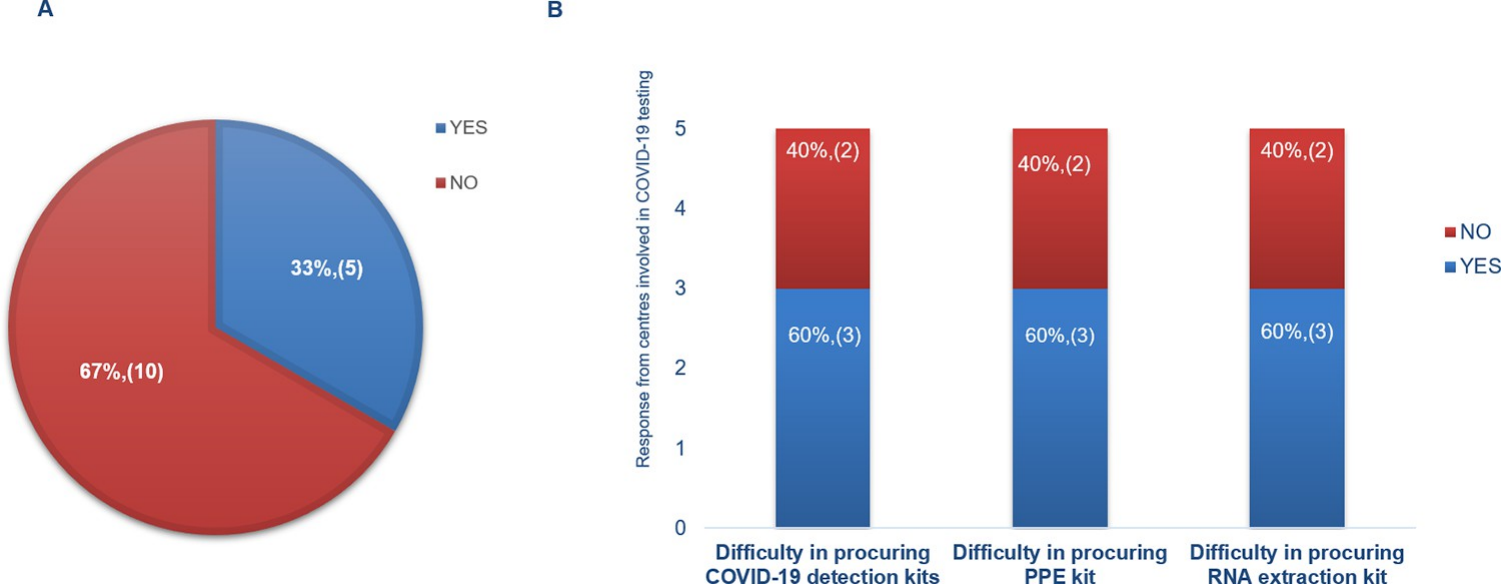
Responses from 15 participants who took part in the survey (A)Participants involved in COVID-19 testing (B) Issues faced by participants in procuring COVID-19 consumables.

The survey data addressed the status of testing laboratories which were involved in COVID-19 testing apart from routine diagnostics and whether adequate facilities were available with them to carry both kinds of diagnostic testing efficiently. Out of 25 centres,15 centres participated in the survey. Among the participants of the survey, 5 centres were involved in COVID-19 testing as a primary responsibility and 10(66%) were indirectly associated with COVID-19 testing facility in their respective premises by means of sharing infrastructure and man power (Fig 1A).

#### 2. Effect on supply of COVID-19 consumables and reagents

Discrete effect of pandemic on diagnostic procedures was noted in the operations of participant centres. Out of 5 centres engaged with COVID-19 testing as primary responsibility, 3(60%) of them faced delays in procuring nucleic acid extraction kits, COVID-19 test reagents and Personal Protective Equipment (PPE) kits (Fig 1B).

#### 3. Effect on diagnostic test requisitions and consumables

**Fig 2:**
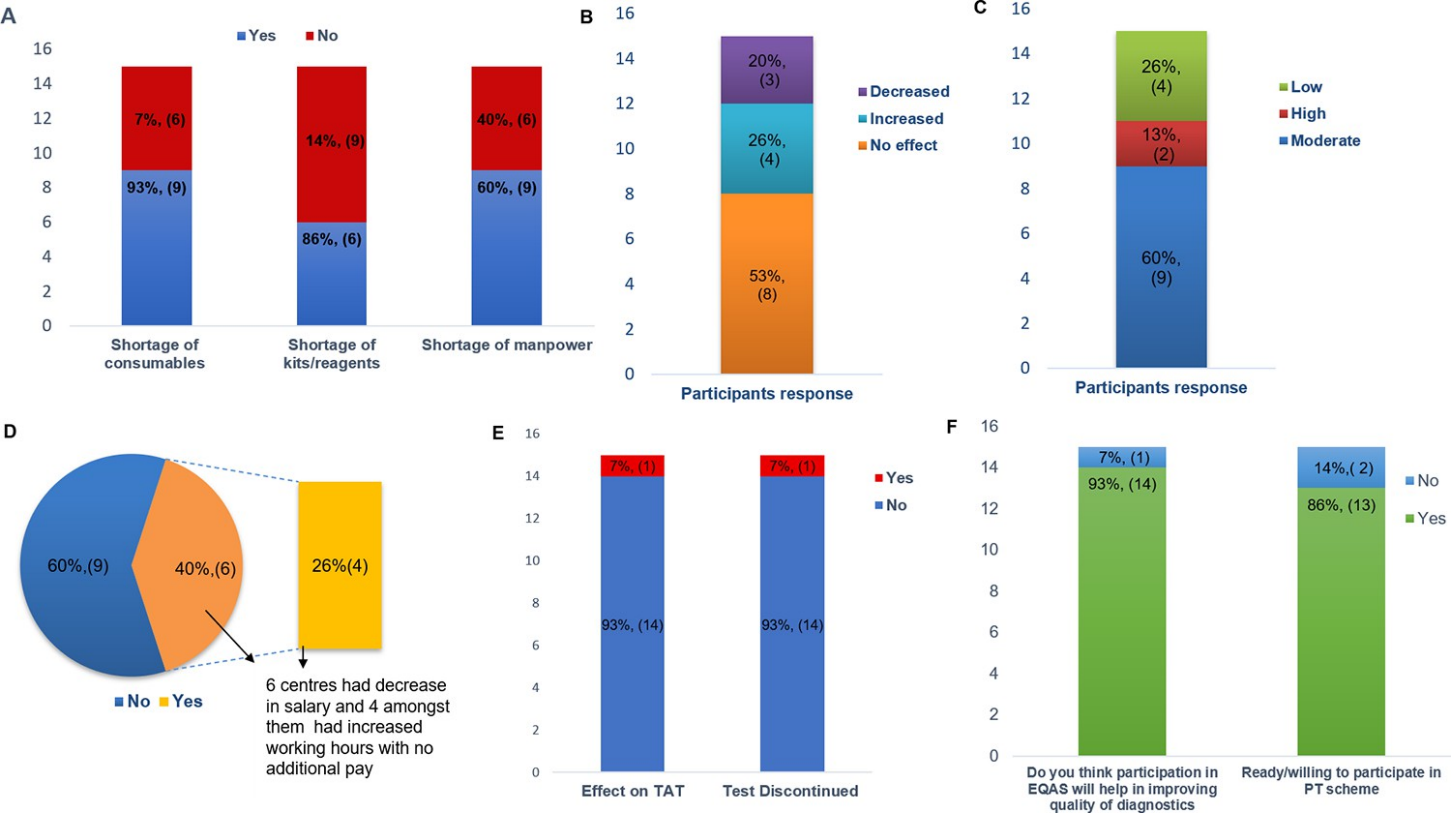
Survey response data from various participating centres depicting the impact of pandemic on routine diagnostics (A)Effect on kits, consumables and manpower (B) Effect on volume of test requisitions (C) Effect on revenue generated from diagnostics (D) Effect on work profile and pay (E) Effect on Turn-around time (TAT) for testing and discontinuation of tests (F) Response for participation in EQAS scheme.

Impact on diagnostic kits shortage and reagents was observed in 6(40%) centres and 9(60%) centres faced issues with supply of consumables such as plastic-wares, gloves, filter tips and surgical masks (Fig 2A)

With respect to impact of COVID-19 on test requisitions, 8(53%) centres had no effect on incoming test requisitions whereas 4(26%) laboratories had increased test numbers and 3(20%) centres had decrease in number of requisitions (Fig 2B).

#### 4. Effect on revenue, remuneration and manpower

In terms of revenue generation of the laboratories from the diagnostic tests only 2 (13%) centres reported about less revenue generation during pandemic whereas 9(60%) centres had moderate effect and 4 (26%) centres had no impact on revenue income (Fig 2C). Likewise, in work profile and pay cuts of personnel, 6 (40%) centres reported to have decrease in salary and 4(26%) amongst them had increased working hours with no additional pay and remaining centres had no impact on the work schedule or on the salary (Fig 2D).

#### 5. Effect on TAT for reporting of test results

With regards to effect of pandemic on TAT for reporting and reasons for test discontinuation, responses from two centres were received. One centre stated about slight delay in diagnostic reporting due to lag in procuring consumables and budget issues whereas the other centre had to discontinue FISH and few immunohistochemistry (IHC) tests citing reasons of extreme shortage of reagents and funds to execute the tests (Fig 2E).

#### 6. Use of alternate quality procedures

**Fig 3:**
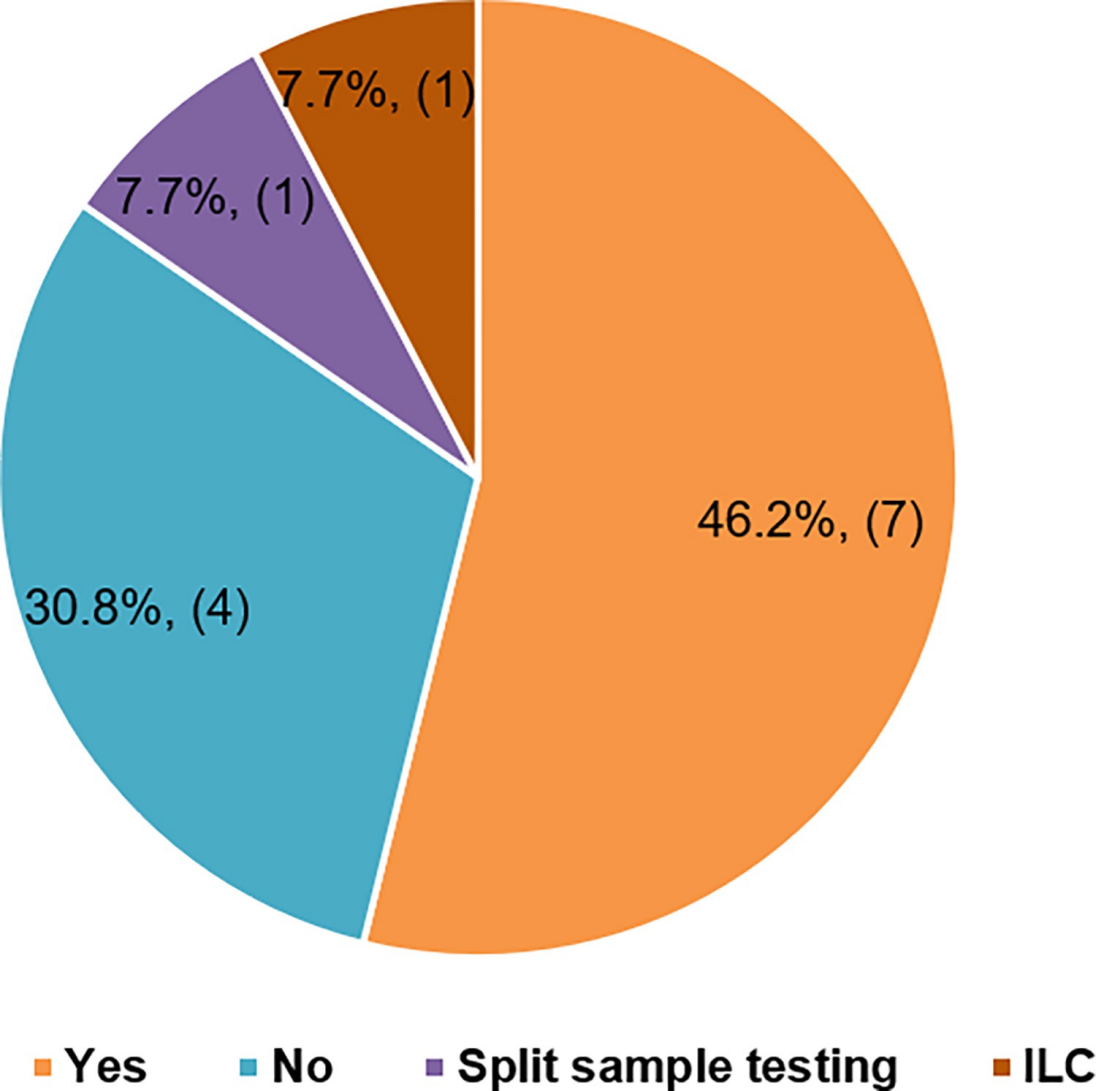
Response to survey conducted on use of alternative quality measures followed by participant laboratories.

Use of alternate assessment procedures (AAP) by participants other than involvement in EQAS (impeded by lockdown) was investigated in the survey and it was observed that 7(46.2%) labs performed AAP such as running known positive controls, validation of results on different platforms or by modification of some steps. Split sample testing and ILC (inter laboratory comparisons) were employed by one centre each and 4 centres did not employ any AAP (Fig 3).

These findings from the survey suggested that the conventional conditions of testing and continual improvement had marginal impact during this pandemic where demand for COVID-19 testing was surging. However, despite such shortcomings, there was a notable response from 13(86%, Fig 2F) centres for willingness to participate in EQAS scheme.

### Performance of participants for FISH *Her2/neu* EQAS cycle

**Fig 4:**
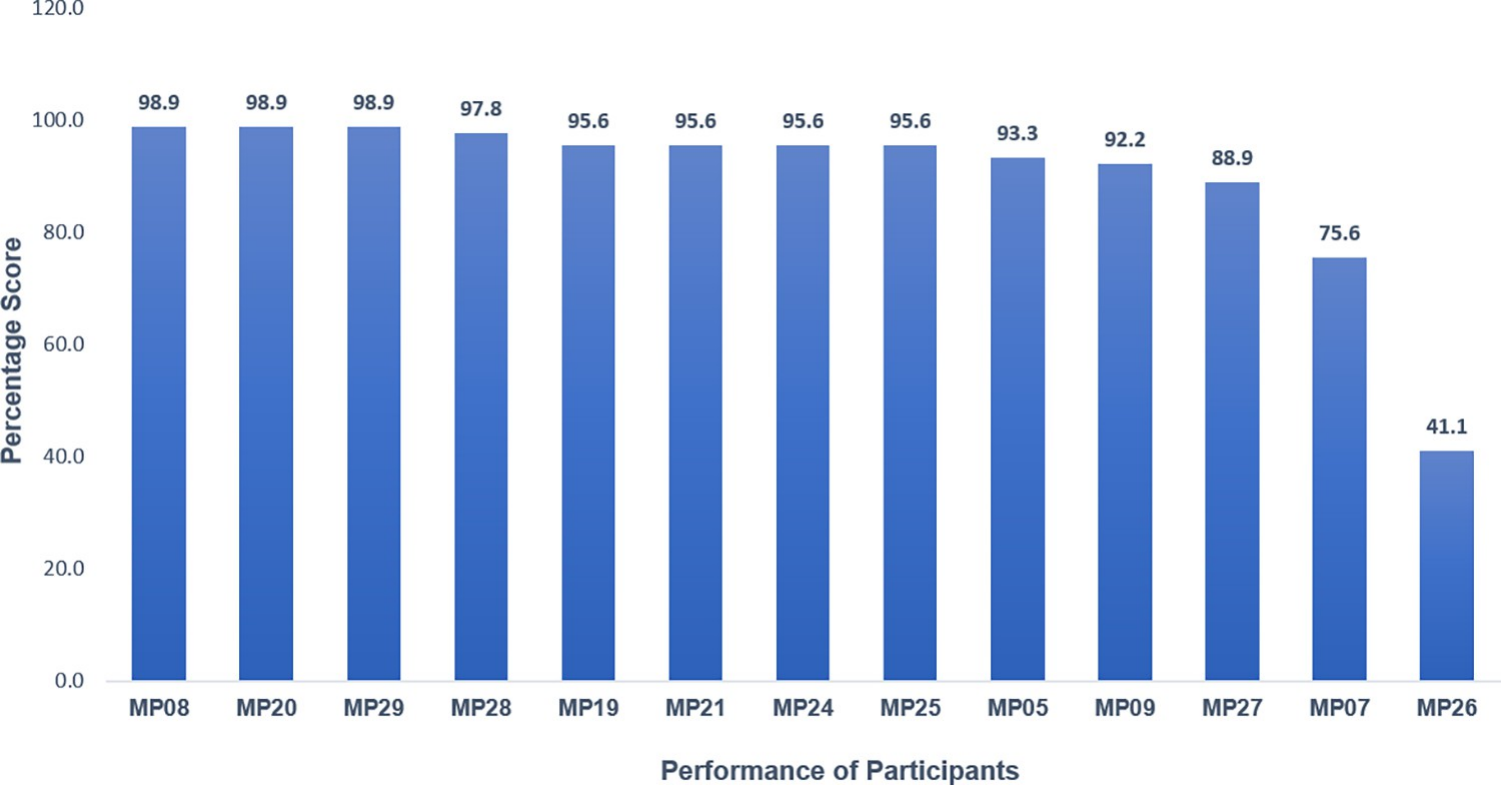
Performance of all participants who participated in the FISH-*Her2/neu* module.

Evaluation of participant results for *Her2/neu* gene amplification test by FISH was based on molecular interpretation of all the samples along with control sample. The performance was assessed for accuracy of testing and prognosis offered based on the findings. Confidential Assessment Report (CAR) of all the participant centres was communicated via online portal system. The competency evaluation of participants for technical skills and reporting was found to be within acceptable limits affirming the reliability and accuracy of reporting by participating centres (Fig 4).

**Fig 5:**
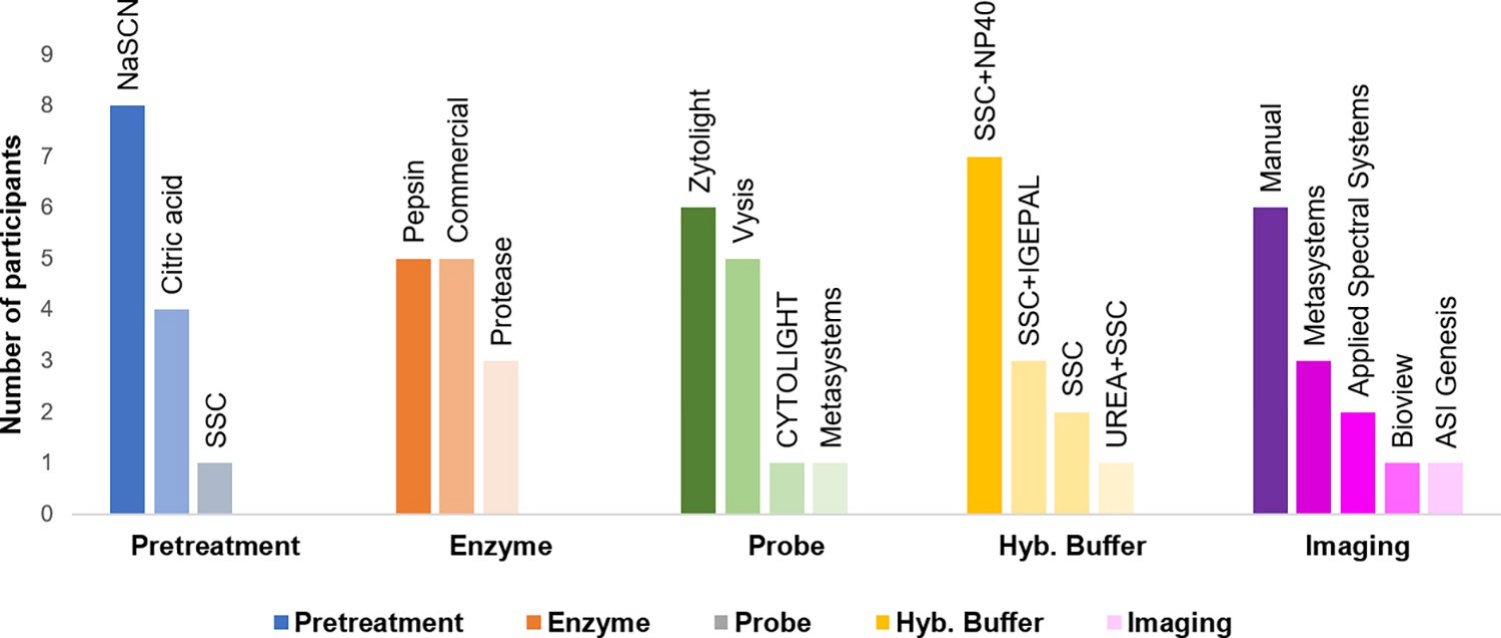
Trend of different methods of pre-treatment, enzymes used, probe used along with hybridization buffer and image analysis software employed by participants for the FISH-*HER2/neu* module.

Moreover, variation in procedures adopted by participants in performing FISH technique had reliable analytical specificity and sensitivity signifying the standards of quality testing by participating centres (Fig 5).

### Participation results of digital EQAS cycle

**Fig 6:**
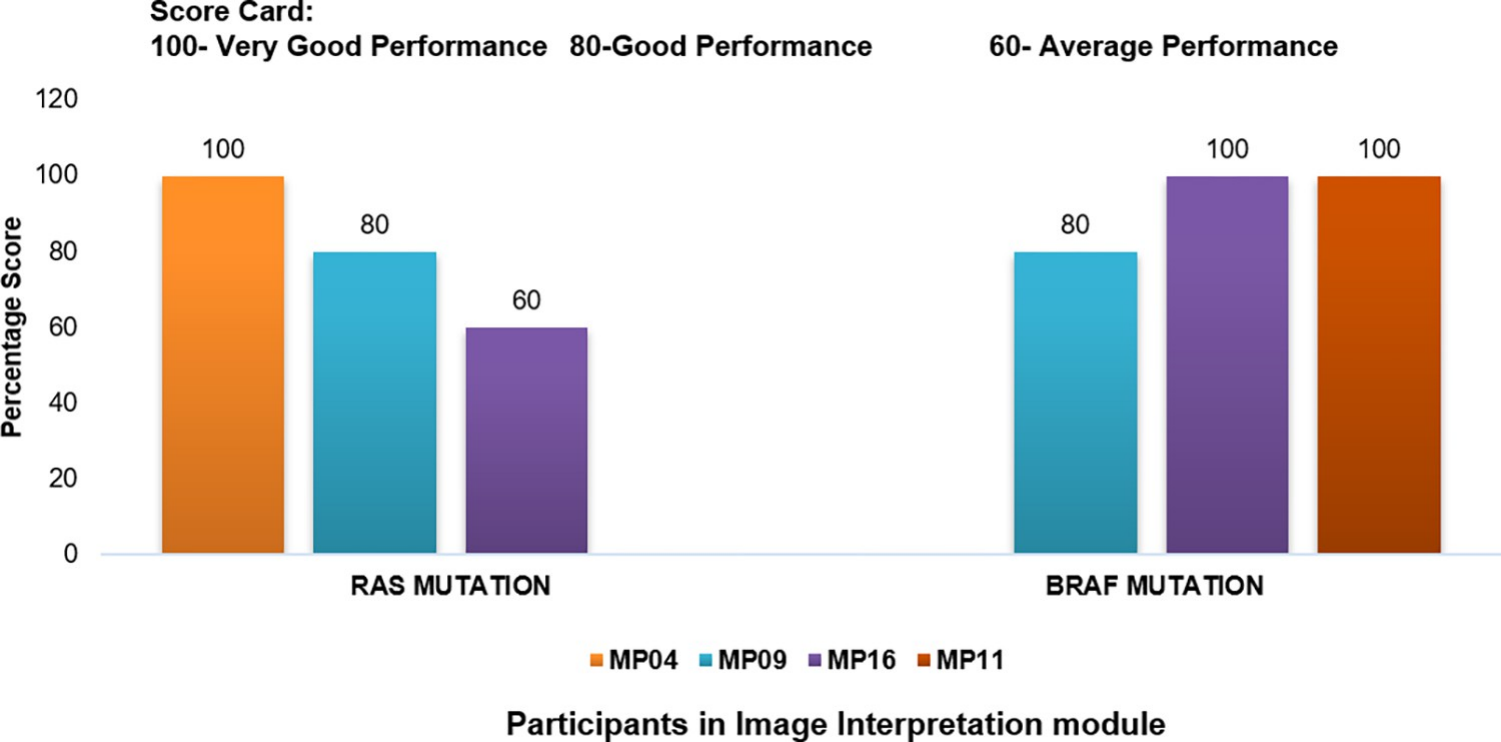
Performance of participants who participated in Digi EQAS module for *RAS* and *BRAF* mutations.

In the Image Interpretation Skill module for identification of *RAS* and *BRAF* mutations, a total of four centres participated, out of which two participated in both modules and two participated either in *RAS or BRAF* mutation respectively. The evaluation of participant results for digital EQAS cycle was based on accurate interpretation of the provided images with correct nomenclature used for mentioning the mutation status of the test sample. Being on virtual platform the evaluation of participant results required shorter time for providing assessment reports. The overall performance showed a range of aptitude level from average to very good performance among participants (Fig 6).

**Fig 7:**
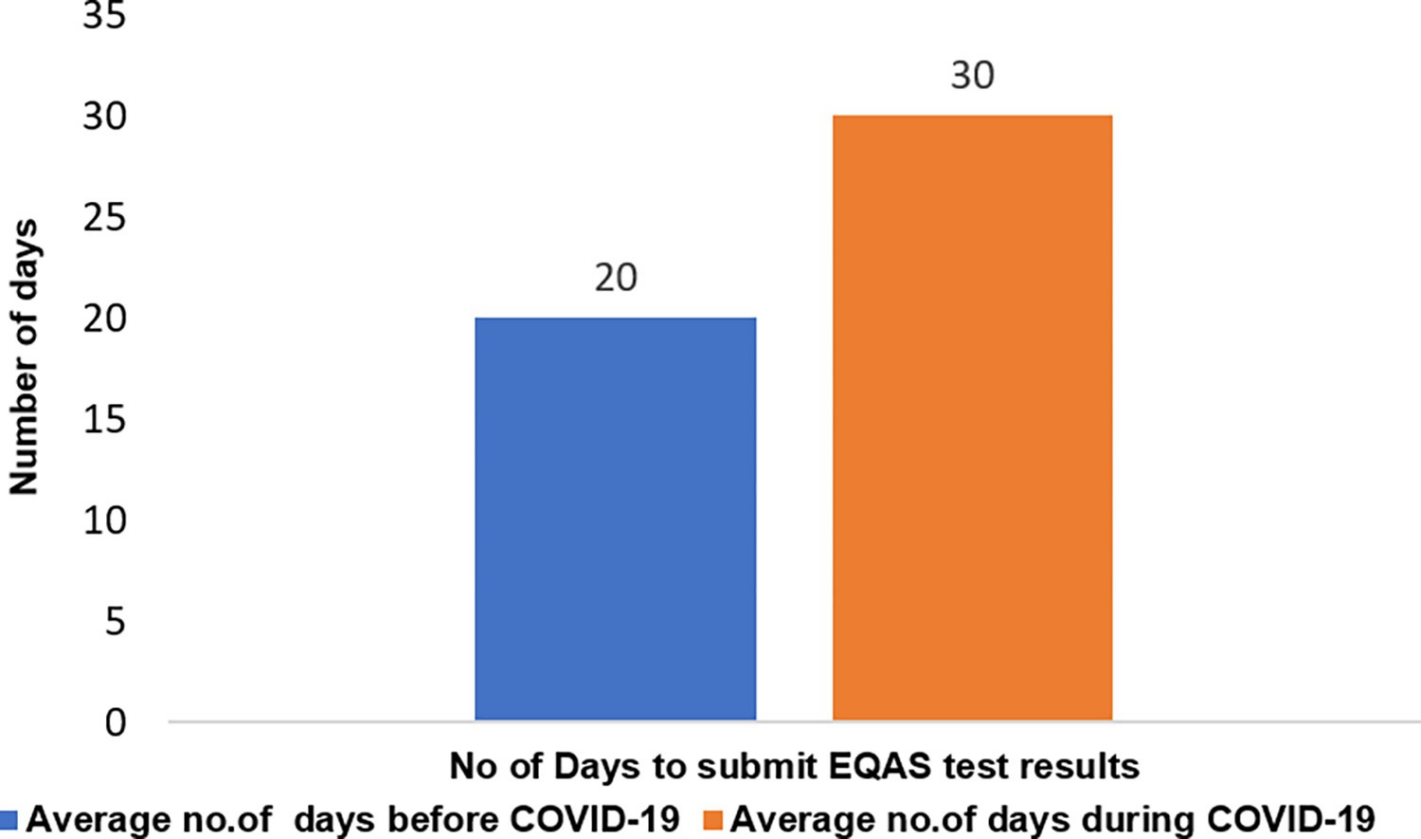
Effect of pandemic on number of days required to submit the test results before and during pandemic.

Additionally, during completion of EQAS cycle, it was observed that the time duration provided for testing of EQAS samples and receiving results (generally 20 days from the receipt of the test material by the participants) from participants for each round of cycle was significantly affected due to the interstate lockdown protocols (Fig 7). This effect was evident from survey data (Fig 2A) where centres faced shortage of kits, reagents and manpower due to engagement in COVID-19 testing.

## Discussion

There is limited information about the challenges faced in managing clinical laboratory services outside of COVID-19 specific diagnostics, although reports are beginning to emerge. The ongoing pandemic is an awakening experience to carry out EQAS service where conventional rulebook is replaced by innovative measures and novel approaches in all spheres of life.

Imposition of lockdown across the country led to impediment of logistics for sending proficiency test materials, impacting the primary role of quality evaluation of EQAS program. Nonetheless, it provided impetus to make use of the secondary objective of PT provider in inquiring into the shortcomings faced during pandemic and ministering necessary guidance.

The survey responses imparted a fair idea on prevailing status of diagnostic procedures carried out in different laboratories for molecular diagnostics (Figs 1 and 2).Employment of AAP in absence of EQAS (due to lockdown and non-participation) by laboratories to verify the acceptability of test performance is a good practice [17]. Dependency on AAP can be complex due to variation in methods, lack of recommendations regarding selection of samples, relaxed and less inadequately defined frequency. Also, there are no well characterized and standard protocols as it is organized differently in each laboratory. [18]. However, the decision to choose enhancement of quality services either by participation in EQAS or AAP lies in the best interest of testing laboratories, where additional considerations such as fiscal issues and awareness regarding available EQAS schemes seem to play a role. Quality of existing molecular diagnostic tests cannot be compromised under the pretext of a pandemic crisis or otherwise as it would directly impact patient care.

Implementation of rotational shifts or staggered duty hours among the technical personnel to minimize social contact, cross-sectoral skill management, positive attitude and the active preparedness among participants to participate in EQAS schemes proved that quality standards can be sustained despite threats posed by the virus.

Routine diagnostic procedures were not affected significantly due to pandemic and the active willingness of participants paved the way to conduct EQAS scheme using reformed approach. In an era of digital pathology and artificial intelligence (AI), remote reporting by pathologists has become a reality with use of high-definition WSI and online cloud based digital software tools for molecular analysis [19]. This concept of virtual reporting was envisaged and effectively utilized to implement Digital EQAS scheme. Digital tools played an instrumental role in demonstrating the importance of information technology in offering PT services. This proved to be a value addition which could assist in remote management of EQAS.

The need of setting up databases for use of alternate protocols and procedures during crisis can help reduce the lag in processing of the samples due to shortage of reagents especially in the presence of heightened pressure of providing test reports on priority. This can be achieved by willingness to actively participate in EQAS programs by testing laboratories, where PT providers like MPQAP, in an advisory role, can aid in data management and monitor the quality change in solid tumour testing over a period of time [15].

## Conclusions

Improved and enhanced quality measures related to solid tumour molecular diagnostic testing should be substantiated by PT programs [20]. Participation in EQAS schemes such as MPQAP offer opportunities for the laboratories to assess their performance vis-a-vis other participating laboratories. Our experience emphasizes that laboratories should participate in PT programs for quality sustenance, monitoring and improvement of testing services irrespective of any circumstances like COVID-19 pandemic where continual support of EQAS providers will be rendered by adopting novel approaches and reformed methods.

## Supporting information

S1 Data (XLSX)

S1 File (PDF)

S2 File (PDF)

S3 File (PDF)

S1 Table Calendar schedule of MPQAP EQAS for the year 2020–2021. (DOCX)
