# Supplementary material for: Impact of COVID-19 on quality checks of solid tumor molecular diagnostic testing-A surveillance by EQAS provider in India
Source: PLoS One. 2022 Sep 22;17(9):e0274089. doi: 10.1371/journal.pone.0274089 (PMC9498956; doi:10.1371/journal.pone.0274089)
Supplement: S1 File — (PDF) [file pone.0274089.s002.pdf]

DIAGNOSTIC LABORATORY SAFETY PRECAUTIONS AND MEASURES IN CONTEXT OF COVID 19 PANDEMIC

INTRODUCTION:

Severe Acute Respiratory Syndrome Coronavirus-2 (SARS-CoV-2), commonly known as 2019 novel coronavirus (COVID-19) has been declared as pandemic by the World Health Organization. In state of absence of vaccines as well as uncertainty of medications to treat or limit the spread and with little scientifically validated information on this novel virus, the laboratories working on patient samples involved in diagnostic testing and having suspected/confirmed positive COVID-19 samples should follow measures and practices as precautionary actions to prevent further spread.

The purpose of this document is to provide an interim guideline on laboratory biosafety (in tune with the international norms) to be followed in handling and processing clinical samples/infectious virus for diagnostic testing.

The basic minimal procedures to be followed are:

1. All procedures must be performed based on risk assessment and only by personnel with demonstrated capability in strict observance to any relevant protocols at all times.
2. It is highly recommended to start local risk assessment for each of the process step, i.e. sample collection, different processes that are planned in the laboratory) and for each of the process step the potential hazards (e.g., aerosol exposure, potential spillage etc.,) have to be considered and assessed with a grade of risk.
3. Non-propagative diagnostic laboratory work (e.g. nucleic acids, sequencing, NAAT, PCR, isolation of antibodies, serum proteins) should be conducted in laboratories with facilities and procedures equivalent to Level II Biosafety Cabinet (BSL-2).

4. Appropriate disinfectants with proven activity against enveloped viruses should be used (e.g. hypochlorite (bleach), alcohol, hydrogen peroxide, quaternary ammonium compounds and phenolic compounds).
5. All technical procedures should be performed with standard operating protocols that minimize the generation of aerosols and droplets.
6. Suspected or confirmed COVID-19 specimens, with appropriate identification labeling, should be stored at a designated place with controlled access to authorized personnel only at 2-8°C depending on the nature of the experiment(s). Extracted nucleic acid samples should be stored at -70° C or lower. All diagnostic laboratories should strictly follow the retention period as per standard guidelines for the samples submitted to them for testing.
7. For the selection of appropriate decontamination and disinfection strategies for biomedical waste treatment and disposal should be in accordance to those mentioned in the "Revised Guidelines for Common Bio-medical Waste Treatment and Disposal Facilities" (2016) developed by Central Pollution Control Board (CPCB).
8. The laboratory waste should be handled like other biohazardous waste as per the DBT notified "Regulations and Guidelines on Biosafety of Recombinant DNA Research and Bio-containment, 2017".
9. The employing authority, through the laboratory director, must take responsibility for ensuring that the health of laboratory personnel is adequately checked and reported.

MPQAP
Molecular Pathology - Quality Assurance Program

STAFF SAFETY GUIDELINES:

WHO provides guidance for the use of PPE, including masks, by health care workers (HCW) in the guidance document-**Advice on the use of masks in the context of COVID-19interim guidance.**

The following are excerpts derived from these documents**:**

Health care workers should:

- Wear a three plysurgical mask when entering a room where patients with suspected or confirmed COVID-19 are admitted.
- Use a particulate respirator at least as protective as a US National Institute for Occupational Safety and Health-certified N95, European Union standard FFP2, or equivalent, when performing or working in settings where aerosol-generating procedures, such as tracheal intubation, non-invasive ventilation, tracheotomy, cardiopulmonary resuscitation, manual ventilation before intubation, and bronchoscopy are performed.
- Place the mask carefully, ensuring it covers the mouth and nose, and tie it securely to minimize any gaps between the face and the mask.
- Avoid touching the mask while wearing it.
- Avoid use of Cotton cloth masks
- Remove the mask using the appropriate technique: do not touch the front of the mask but untie it from behind.
- Discard single-use masks after each use and dispose of them immediately upon removal.
- Replace masks as soon as they become damp with a new clean, dry mask.
- After removal or whenever a used mask is inadvertently touched, clean hands using an alcohol-based hand rub or soap and water if hands are visibly dirty.
- Do not re-use single-use masks.
- Avoid contact of gloved hands with the face.

Inside the laboratory

i. Ensure prior to entry into the laboratory, supplies of laboratory equipment and consumables, including reagents, PPE and disinfectants, are sufficient and appropriate for the activities being performed.

ii. Refrain from using mobile electronic devices (for example, mobile telephones, tablets, laptops, flash drives, memory sticks, cameras and/or other portable devices

MPQAP
Molecular Pathology - Quality Assurance Program
A program by National Cancer Grid

including those used for DNA/RNA sequencing) when not specifically required for the laboratory procedures being performed.

iii. Keep mobile electronic devices in areas where they could not easily become contaminated or act as a fomite for infection. Where close proximity of such devices to biological agents is unavoidable, ensure they are either protected by a physical barrier or decontaminated before leaving the laboratory.

PERSONAL PROTECTIVE EQUIPMENT (PPE)

i. Laboratory coats must be used in laboratories to prevent personal clothing from getting splashed or contaminated by biological agents.

ii. Where possible, the fabric of the laboratory coat should be splash resistant and overlap to provide a solid front. Laboratory coats must only be worn in designated areas.

iii. Appropriate disposable gloves must be worn for all procedures that may involve planned or inadvertent contact with blood, body fluids and other potentially infectious materials.

iv. Gloves must not be disinfected or reused as exposure to disinfectants and prolonged wear will reduce the integrity of the glove and decrease protection to the user.

MPQAP
A program by National Cancer Grid

USING PPE GEARS:

- All personnel at the testing center must receive comprehensive training and demonstrate competency in using PPE.
- Leave all personal belongings including gadgets, watches, rings etc, outside. Tie long hair back. Facial hair – beards, mustache etc – should be shaved.
- Wash your hands with soap and water (recommended) or wipe with 70% alcohol before entering the facility.
- Allow personnel at least 30min to don and doff the gear. It should be done slowly and mindfully. An onsite manager must be present at all times. Preferably, a trained observer must supervise each step of every PPE donning and doffing.

Donning and Doffing PPE

- Enter the PPE donning area. Unpack PPE kit (surgeon gown, N-95 mask, Head cover, goggles, shoe cover and a pair of gloves). If gloves are powdered, do not use them for molecular biology work; use purple nitrile gloves instead.
- Follow the order to wear: gown→gloves→N95→goggles→disinfect hands.
- Wear double gloves while handling pathogens.
- In the event of a significant splash, the personnel should immediately move to the doffing area to remove PPE.
- For doffing off discard outer pair of gloves first.
- Remove PPE in this order: googles → gown → mask→ gloves.
- Ensure each time it is discarded in the appropriate waste bin.
- Once you exit PPE doffing area, wash hands thoroughly.

COLLECTION AND HANDLING OF SPECIMENS:

i. All specimens collected for laboratory investigations should be regarded as potentially infectious.

ii. Wear disposable gloves at all times when handling specimens.

iii. Ensure that all personnel who transport specimens are trained in safe handling practices and spill decontamination procedures.

iv. Place specimens for transport in leak-proof specimen bags (secondary containers) that have a separate sealable pocket for the specimen (a plastic biohazard specimen bag), with the patient's label on the specimen container (the primary container), and a clearly written laboratory request form.

v. Ensure that laboratories in health care facilities adhere to appropriate biosafety practices and transport requirements, according to the type of infectious agents being handled; deliver all specimens by hand whenever possible.

vi. DO NOT use pneumatic-tube systems to transport specimens; document clearly each patient's full name, date of birth and "suspected COVID-19" on the laboratory request form.

vii. Notify the laboratory as soon as possible that the specimen is being transported.

MPQAP
Molecular Pathology - Quality Assurance Program
A program by National Cancer Grid

ROUTINE DIAGNOSTIC PROCEDURES:

i. When handling and processing specimens, "core requirements" (CR), including good microbiological practice and procedure (GMPP), should be followed at all times. Refer: **Laboratory biosafety guidance for novel corona virus (2019-nCoV): Interim Recommendations**(https://www.who.int/docs/defaultsource/coronaviruse/laboratory-biosafety-novel-coronavirus-version-1-1.pdf?sfvrsn=912a9847_2)

ii. Local risk assessment should be conducted to determine the use of PPE for performing technical procedures that will be performed outside BSC such as procedures that can cause splashes, grossing, loading and unloading of sealed centrifuge cups, grinding, blending, vigorous shaking or mixing, sonic disruption, opening of containers of infectious materials whose internal pressure may be different from the ambient pressure.

iii. In conditions of performing such technical procedures laboratory workers should wear protective equipment including disposable gloves along with solid front or wrap-around gowns, scrub suitsor coveralls with sleeves that fully cover the forearms, head coverings, shoe covers or dedicated shoes, eye protection (goggles or face shield).

iv. Shield or otherwise protect the mouth, eyes and face using surgical masks and googles during operation where splashes may occur to have respiratory protection.

v. For work needing scissors, use scissors with blunt or rounded ends in preference to those with pointed ends.

vi. Handle all sharps and needles, if necessary, with care so as to prevent injury and injection of biological agents.

vii. Use ampoule openers for safe handling of ampoules. Minimize the risk associated with the use of syringes or with needles

viii. Never re-cap, clip or remove needles from disposable syringes.

ix. Dispose of any sharps materials (for example, needles, needles combined with syringes, blades, broken glass) in puncture-proof or puncture-resistant containers fitted with sealed covers.

x. Discard specimens and cultures for disposal in leakproof containers with tops appropriately secured before disposal in dedicated waste containers.

xi. Consider opening tubes with disinfectant soaked pad/gauze.

xii. Centrifugation of specimens should be performed using sealed centrifuge rotors or sample cups. These rotors or cups should be loaded and unloaded in a BSC.

MPQAP
Molecular Pathology - Quality Assurance Program
A program by National Cancer Grid

xiii. A dedicated hand-wash sink should be available in the laboratory.

xiv. Decontaminate work surfaces with a suitable disinfectant at the end of the work procedures and if any material is spilled or obviously contaminated. Ensure the disinfectant is efficacious against the pathogen being handled and is left in contact with infectious waste materials for sufficient time to effect complete inactivation.

xv. After use, bench spaces should be cleaned with 10% sodium hypochlorite (followed by sterile water to remove residual bleach), 70% ethanol, or a validated commercially available DNA-destroying decontaminant.

xvi. Do not clean the vortex mixer and centrifuge machine with sodium hypochlorite; instead, wipe down with 70% ethanol and expose to UV light, or use a commercial DNA-destroying decontaminant.

ROUTINE DECONTAMINATION OF LAB AREA

i. Virus aerosols are notorious for sticking around at several surfaces. It is important therefore to have a fixed decontamination schedule, preferably once every week which includes wiping down all surfaces including door knobs.

ii. Maintain cleaning protocol sheets with log for all the workstations in the Pre PCR and Post PCR area. Follow the procedure: Wipe with 4% hypochlorite followed by 70% alcohol.

iii. For BSC, turn on UV for 30 minutes before and after each batch of samples is processed.

iv. Periodically perform swab test from all the surface areas (Pre and Post PCR areas) to ensure that these surfaces are free of any aerosol generated contaminants which could be major risk for laboratory contamination.

MPQAP
A program by National Cancer Grid

SPILL CLEAN UP PROCEDURE:

Written procedures for cleaning and decontaminating spills must be developed for the laboratory and followed by suitably trained personnel.

Spill kits, including disinfectant, must be easily accessible to personnel.

In the event of a spill of infectious or potentially infectious material, the following spill clean-up procedure should be used:

i. Wear disposable gloves and protective clothing, including face and eye protection if required.
ii. Cover the spill with cloth or paper towels to contain it.
iii. Pour an appropriate disinfectant over the paper towels and the immediately surrounding area (generally, 5% bleach solutions are appropriate).
iv. Apply disinfectant concentrically beginning at the outer margin of the spill area, working toward the center.
v. After the appropriate amount of time (e.g. 30 min), clear away the materials; glass fragments should be handled with forceps.Clean and disinfect the area of the spillage (if necessary, repeat steps ii-v).
vi. Dispose of contaminated materials into a leakproof, puncture-resistant waste disposal autoclave able bag.
vii. If laboratory forms or other printed or written matter are contaminated, the information should be copied onto another form and the original should be discarded.
viii. After successful disinfection, inform the competent authority that the site has now been decontaminated

MPQAP
Molecular Pathology - Quality Assurance Program
A program by National Cancer Grid

MANAGEMENT OF BIOMEDICAL WASTE

i. Keep separate color-coded bins/bags/containers in wards and maintain proper segregation of waste as per BMWM (Biomedical Waste Management) Rules, 2016 as amended and CPCB (Central Pollution Control Board) guidelines for implementation of BMW Management Rules.

ii. Following color coded bags should be used for disposal of different categories of biomedical waste generated:

CATEGORY	TYPE OF WASTE
YELLOW	a. Human Anatomical Waste, Animal Anatomical Waste,<br>b. Items contaminated with blood, body fluids like dressings, plaster casts, cotton swabs and bags containing residual or discarded blood and blood components.<br>c. Discarded linen, mattresses, beddings contaminated with blood or body fluid, routine mask & gown.<br>d. Pharmaceutical waste like antibiotics, cytotoxic drugs including all items contaminated with cytotoxic drugs along with glass or plastic ampoules, vials etc.<br>e. Liquid waste generated due to use of chemicals in production of biological and used or discarded disinfectants, Silver X - ray film developing liquid, discarded Formalin, infected secretions, aspirated body fluids, liquid from laboratories an d floor washings, cleaning, house - keeping and disinfecting activities etc<br>f. Blood bags, Laboratory cultures, stocks or specimens of microorganisms, live or attenuated vaccines, human and animal cell cultures used in research, production of biological, residual toxins, dishes and devices used for cultures
RED	Wastes generated from disposable items such as tubing, bottles, intravenous tubes and sets, catheters, urine bags, syringes without needles, fixed needle syringes with their needles cut, vaccutainers and gloves

MPQAP
Molecular Pathology - Quality Assurance Program
A program by National Cancer Grid

**WHITE**	Waste Sharps including metals Needles, syringes with fixed needles, needles from needle tip cutter or burner, scalpels, blades, or any other contaminated sharp object that may cause puncture and cuts. This includes both used, discarded and contaminated metal sharps
**BLUE**	Broken or discarded and contaminated glass including medicine vials and ampoules except those contaminated with cytotoxic wastes.

iii. Report opening or operation of COVID-19 ward and COVID ICU wardto SPCBs (State Pollution Control Board) and respective CBWTF (Common Bio Medical Waste Treatment Facility) located in the area.

iv. As precaution double layered bags (using 2 bags) should be used for collection of waste from COVID-19 isolation wards so as to ensure adequate strength and no-leaks.

v. It is mandatory for bags/containers used for collecting biomedical waste from COVID-19 wards, should be labelled as "COVID-19 Waste".

vi. Use a dedicated collection bin and trolleys labelled as "COVID-19" to store COVID-19 waste and keep separately in temporary storage room prior to handing over to authorized staff of CBWTF.

vii. The (inner and outer) surface of containers/bins/trolleys used for storage of COVID-19 waste should be disinfected with 1% sodium hypochlorite solution daily.

viii. Collect used PPEs such as goggles, face-shield, splash proof apron, Plastic Coverall, Hazmet suit, nitrile gloves into RED bag.

ix. Collect used masks (including triple layer mask, N95 mask, etc.), head cover/cap, shoe-cover, disposable linen Gown, non-plastic or semi-plastic coverall in YELLOW bags.

x. Pre-treat viral transport media, plastic vials, vacutainers, eppendorf tubes, plastic cryovials, pipette tips should be disposed in RED bags.

xi. PPEs used and other contaminated waste generated from patients or by COVID-19 Waste handlers and Pathologists shall be stored separately in YELLOW bag shall be Pre-treated with Autoclaving / Microwaving before transfer to temporary storage area and then hand over to Common treatment Facility in YELLOWColored bags with specific marking as "COVID-19 Waste".

MPQAP

REFERENCES:

1. Interim Laboratory Biosafety Guidelines for Handling and Processing Specimens Associated with Coronavirus Disease 2019 (COVID-19) https://www.cdc.gov/coronavirus/2019-ncov/lab/lab-biosafetyguidelines.html

2. Laboratory biosafety guidance for novel coronavirus (2019-nCoV): Interim Recommendations https://www.who.int/docs/default-source/coronaviruse/laboratory-biosafety-novel-coronavirus-version-1-1.pdf?sfvrsn=912a9847_2

3. Interim guidelines for laboratory biosafety to handle COVID-19 specimen for R & D purpose- http://dbtindia.gov.in/sites/default/files/OM_Interim_Guidance_COVID.pdf

4. Advice on use of masks in context of COVID-19-Interim guidelines- https://www.who.int/publications-detail/advice-on-the-use-of-masks-in-the-community-during-home-care-and-in-healthcare-settings-in-the-context-of-the-novel-coronavirus-(2019-ncov)-outbreak

5. Guidelines for Handling, Treatment, and Disposal of Waste Generated during Treatment/Diagnosis/ Quarantine of COVID-19 Patients- Central Pollution Control Board- http://www.mpcb.gov.in/sites/default/files/CPCB-BMW-25032020.pdf

6. Guidelines for Management of Healthcare Waste as per Biomedical Waste Management Rules, 2016- http://www.mpcb.gov.in/sites/default/files/biomedicalwaste/ManagementofHealthcareWaste03032020.pdf

7. Laboratory Biosafety Manual - Third EditionWHOhttps://www.who.int/csr/resources/publications/biosafety/Biosafety7.pdf?ua=1

MPQAP
Molecular Pathology - Quality Assurance Program
A program by National Cancer Grid
