# Supplementary material for: Impact of COVID-19 on quality checks of solid tumor molecular diagnostic testing-A surveillance by EQAS provider in India
Source: PLoS One. 2022 Sep 22;17(9):e0274089. doi: 10.1371/journal.pone.0274089 (PMC9498956; doi:10.1371/journal.pone.0274089)
Supplement: S2 File — (PDF) [file pone.0274089.s003.pdf]

IMPACT OF COVID ON DIAGNOSTIC TESTS

SURVEY BY MOLECULAR PATHOLOGY QUALITY ASSURANCE PROGRAM

* Required

1. Email *

2. Do you wish to participate in the below survey and offer consent for the information provided to MPQAP? *

Mark only one oval.

- Yes
- No

3. Is your lab or department involved in COVID testing? *

Mark only one oval.

- YES *Skip to question 4*
- NO *Skip to question 8*

Lab involved in COVID testing

4. If yes then *

Mark only one oval.

- Directly (prime lab of COVID testing)
- Indirectly (neighboring/other lab involved in testing)

5. Method of COVID testing *

Mark only one oval.

- Antigen-Antibody Based
- Real Time PCR based

6. Average number of COVID Tests performed daily *

7. State the level of difficulties faced for the following *

Mark only one oval per row.

	Normal	Difficult	Very Difficult	Only short delay
Procurement of COVID test reagents	⬭	⬭	⬭	⬭
RNA extraction reagents	⬭	⬭	⬭	⬭
Personal Protective Equipment	⬭	⬭	⬭	⬭

Effect of pandemic

8. How has the pandemic affected the routine diagnostic testing procedures? *

Mark only one oval.

- ⬭ Not that much
- ⬭ Moderate
- ⬭ Highly

9. State the level of difficulty faced for diagnostic procedures during the COVID situation *

Mark only one oval per row.

	Increased	Decreased	Nearby to Normal
Volume of diagnostic test requisitions	◯	◯	◯

10. Whether the TAT for report affected for diagnostic testing- *

Mark only one oval.

- ◯ Yes *Skip to question 11*
- ◯ No *Skip to question 13*
- ◯ Other: ______________________

Effect on TAT

11. If yes, then average delay in reporting days

____

12. Reason for delay in TAT *

Check all that apply.

- [ ] Staff quarantine
- [ ] Shortage of reagents/kits
- [ ] Breakdown of equipment/instrument/lag in maintenance
- [ ] Lack of staff
- [ ] Insufficient funds/budget issues
- [ ] Global issue on analysis network/platform

Other: [ ] ______________________

Shortages

13. Was there shortage of technical staff for performing routine diagnostic test *

Mark only one oval.

( ) Yes *Skip to question 14*

( ) No *Skip to question 15*

Staff shortage

14. If yes, then how has the working profile affected *

Mark only one oval per row.

	Increased	Decreased	No change
Number of Shifts	⬭	⬭	⬭
Working hours	⬭	⬭	⬭
Income	⬭	⬭	⬭

Reagents

15. Any shortage of reagents/kits faced *

Mark only one oval.

⬭ Yes *Skip to question 16*

⬭ No *Skip to question 17*

Shortage of reagent

16. If yes then name of reagent/kits

____

Consumables

17. Shortage of consumables if any *

Check all that apply.

- [ ] Plasticware
- [ ] Filter tips
- [ ] Surgical Masks
- [ ] Powder-free Gloves
- [ ] Head-cap
- [ ] Sanitizers/Alcohol
- [ ] None

18. Any change or modification from current diagnostic testing protocols of testing

Mark only one oval.

- ( ) Yes *Skip to question 19*
- ( ) No *Skip to question 20*

Modification

19. If yes then what modifications

____

Method of reporting

20. Any change in method of reporting, E.g.- Use of cloud tools, digital images, rotational shift for pathologists

Mark only one oval.

- Yes *Skip to question 21*
- No *Skip to question 22*
- Other: ______________________

Reporting

21. If yes then in what way

____

Discontinuation

22. Any test discontinued due to lack of manpower or reagents *

Mark only one oval.

- Yes *Skip to question 23*
- No *Skip to question 24*

Reason

23. If yes then name of test and reason for discontinuation

Mention Name of the test in "Others" section

Check all that apply.

- [ ] Shortage of technical staff due to involvement in COVID testing
- [ ] Extreme shortage/delay in procuring reagents/kits
- [ ] Insufficient funds/budget issues
- Other: [ ] ______________________

Revenue and quality

24. Impact on revenue generated from diagnostic test *

Mark only one oval.

- ( ) Low
- ( ) Moderate
- ( ) High

25. Has the quality procedures of diagnostic testing affected

Mark only one oval.

- Yes
- No
- Not Sure

26. Do you think participation in EQA program will help in improving quality of ongoing diagnostic services?

Mark only one oval.

- Yes
- No
- Maybe
- Likely

27. Have you participated/willing to participate in EQA program during pandemic?

Mark only one oval.

- Yes
- No
- Maybe

28. Any alternate quality control measures followed in your lab if not participated in EQAs

Mark only one oval.

- Yes *Skip to question 29*
- No
- Other: ____________

QC method

29. If yes, then describe in brief about the method-

This content is neither created nor endorsed by Google.

Google Forms
