# Supplementary material for: Impact of COVID-19 on quality checks of solid tumor molecular diagnostic testing-A surveillance by EQAS provider in India
Source: PLoS One. 2022 Sep 22;17(9):e0274089. doi: 10.1371/journal.pone.0274089 (PMC9498956; doi:10.1371/journal.pone.0274089)
Supplement: S3 File — (PDF) [file pone.0274089.s004.pdf]

Gene Sequencing RAS Interpretation Run 1

INSTRUCTIONS

- Try to attempt all 5 questions
- Five images will be provided in the next section
- Write the answer in the column provided below the image and mention the mutation in HGVS format

[Eg- HRAS Exon 2 c.182A>G (p.Q61R) or HRAS c.182A>G (p.Q61R) or HRAS p.Q61R (c.182A>G)]

Limit of sensitivity <20% for image analysis is considered as noise.

- You can submit your response only once.

You can also download the image using right click option.

- Multiple entries from same participant will not be considered.

Last Day to submit response is 26th January 2021.

* Required

1. Email *

2. Centre Name and Centre Code *

Question 1

3. MPSS04 R1 2 points

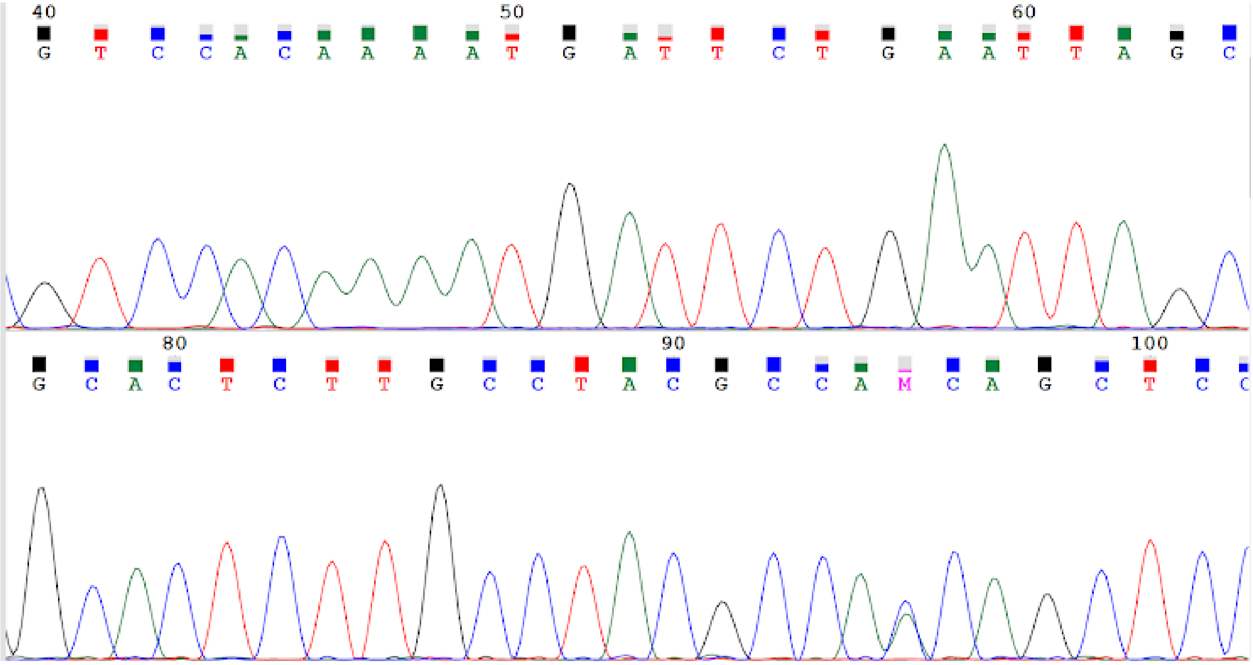

QUESTION 2

4. MPSS04 R2 2 points

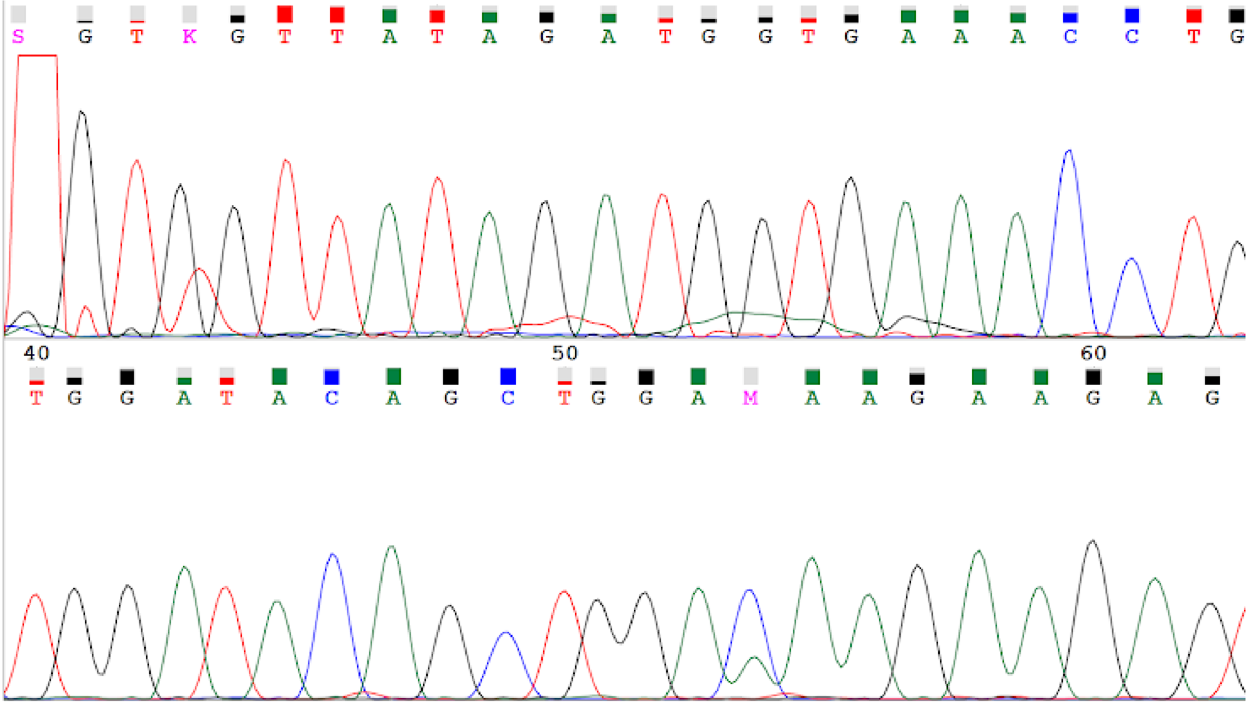

QUESTION 3

5. MPSS04 R3 2 points

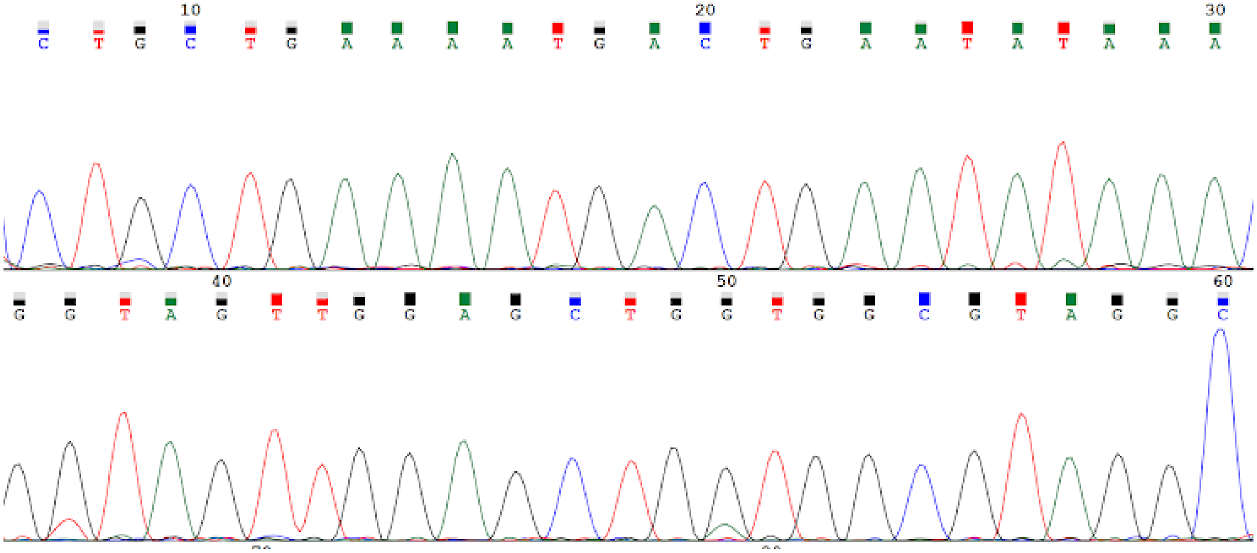

QUESTION 4

6. MPSS04 R4 2 points

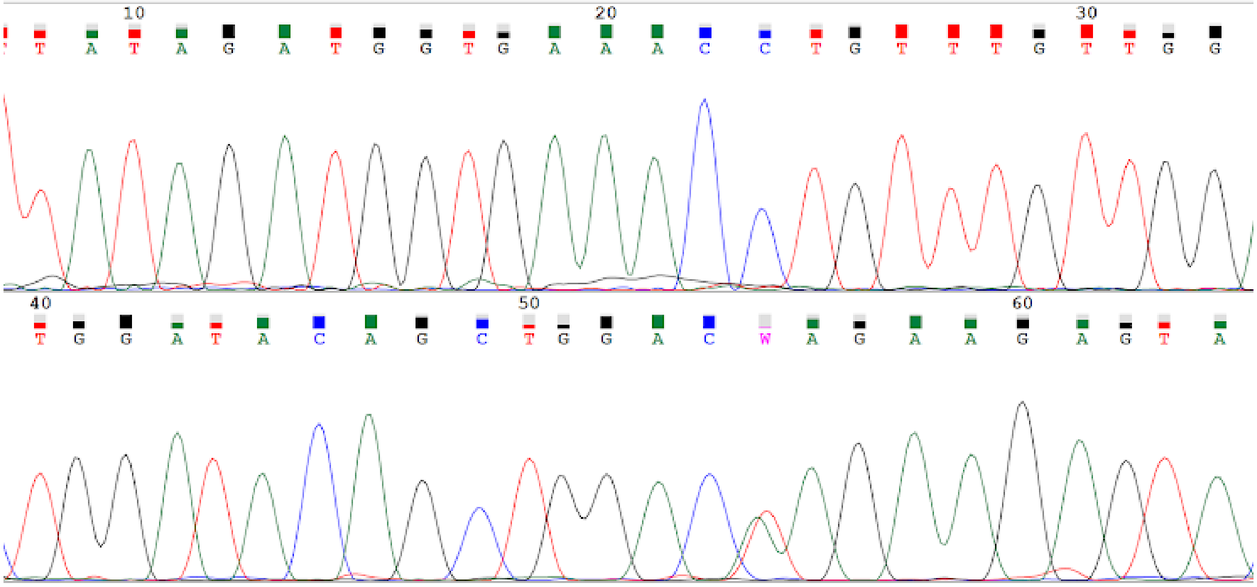

QUESTION 5

7. MPSS04 R5 2 points

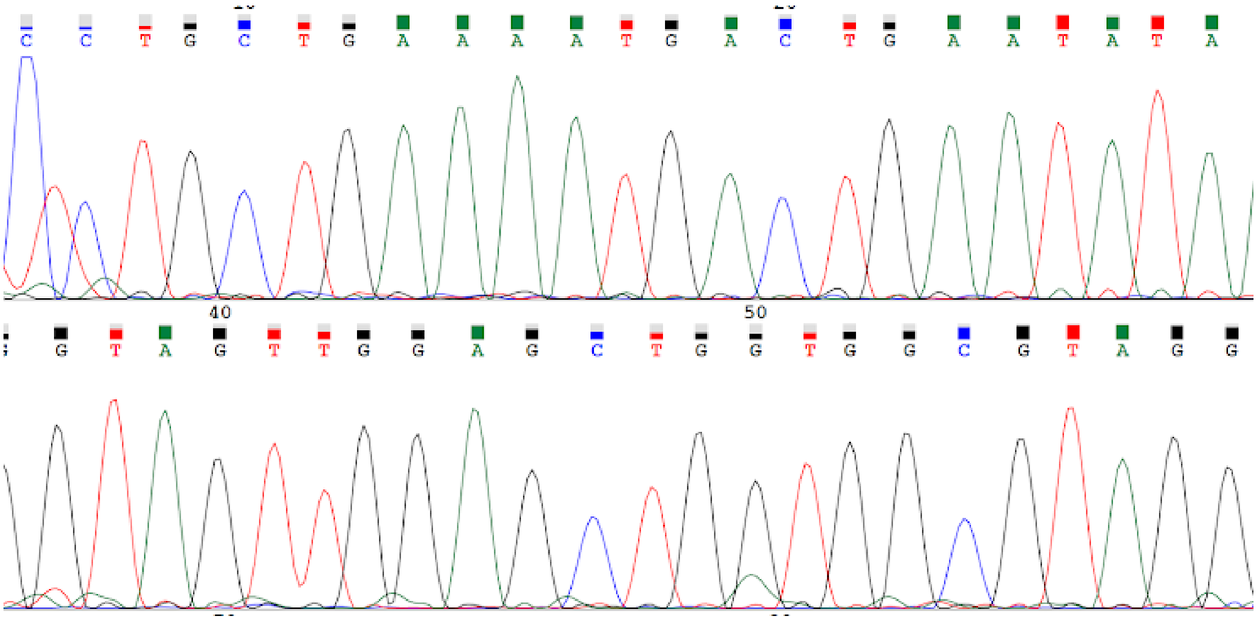

This content is neither created nor endorsed by Google.

Google Forms

Gene Sequencing BRAF Interpretation Run 1

INSTRUCTIONS

- Try to attempt all 5 questions
- Five images will be provided in the next section
- Write the answer in the column provided below the image and mention the mutation in HGVS format

[Eg- BRAF EXON 15 p.V600E/ BRAF (c.1799T>A)]

Limit of sensitivity <20% for image analysis is considered as noise.

- You can submit your response only once.

You can also download the image using right click option.

- Multiple entries from same participants will not be considered.

Last Day to submit response is 26th January 202.

---

* Required

1. Email *

2. Centre Name and Centre Code *

QUESTION 1

3. MPSS01 B1 — 2 points

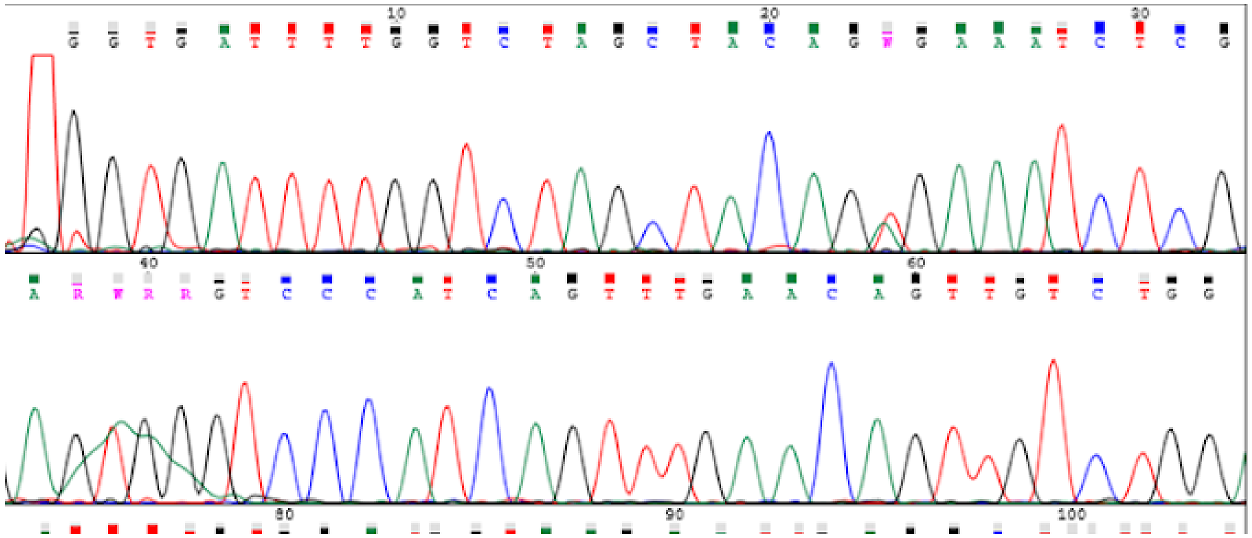

QUESTION 2

4. MPSS01 B2 2 points

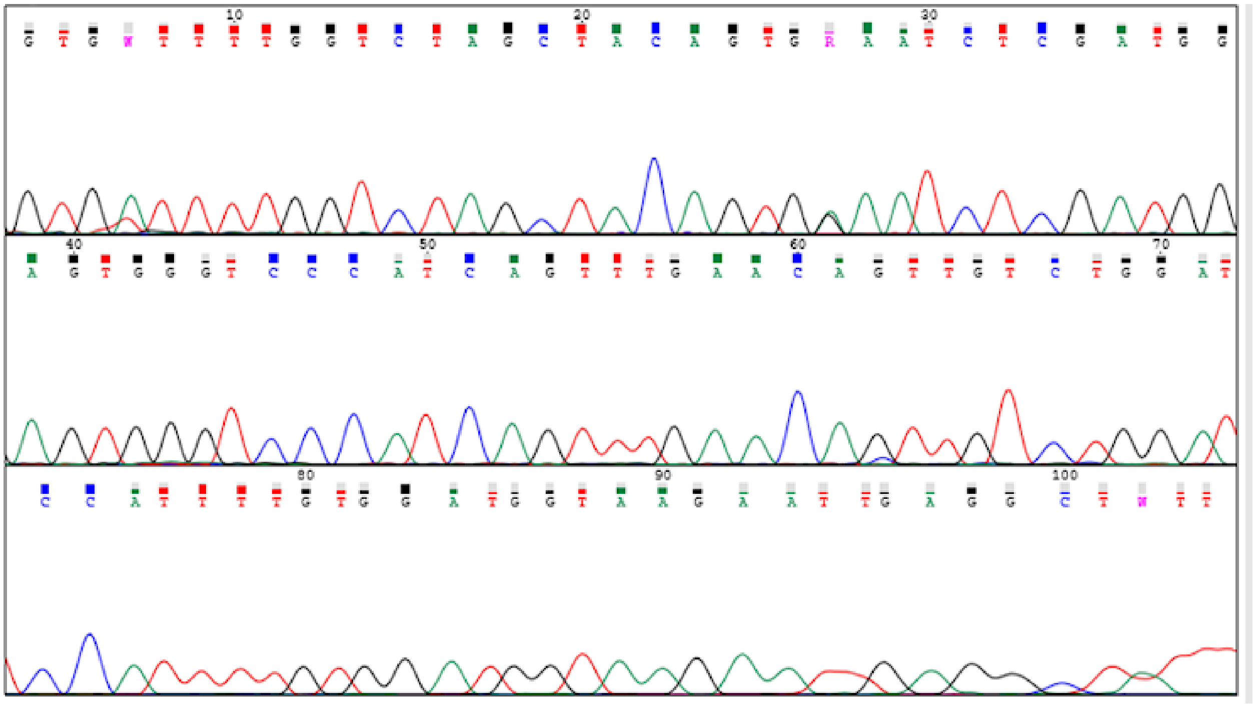

QUESTION 3

5. MPSS01 B3 2 points

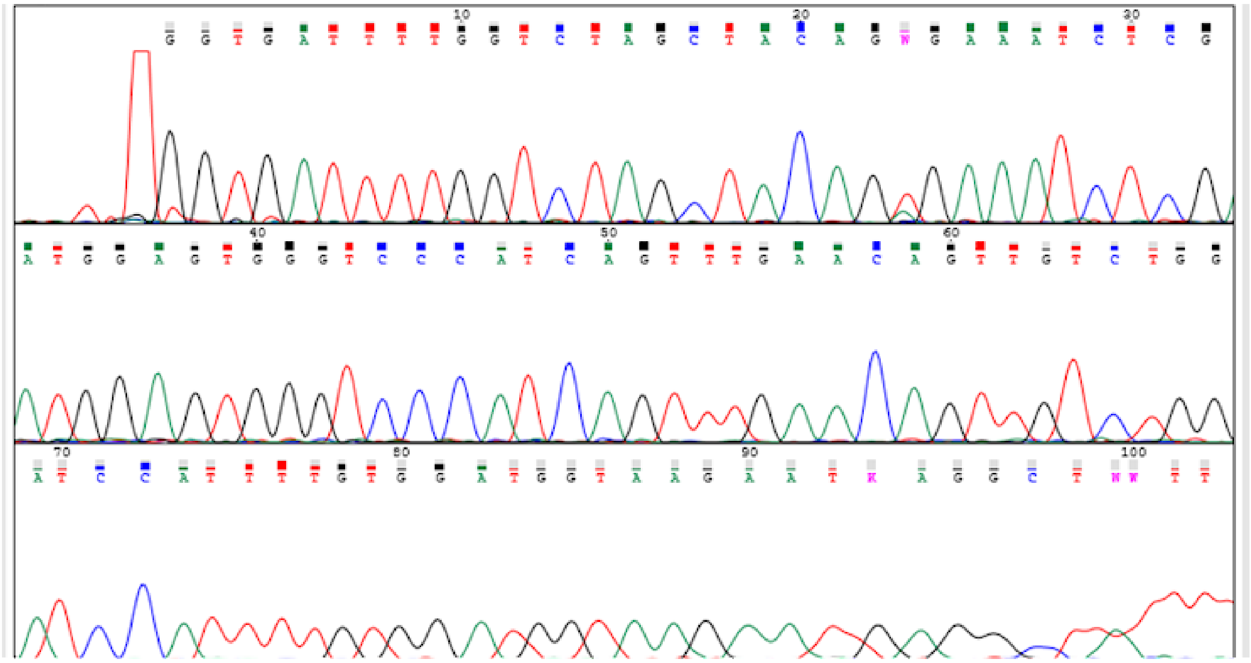

QUESTION 4

6. MPSS01 B4 — 2 points

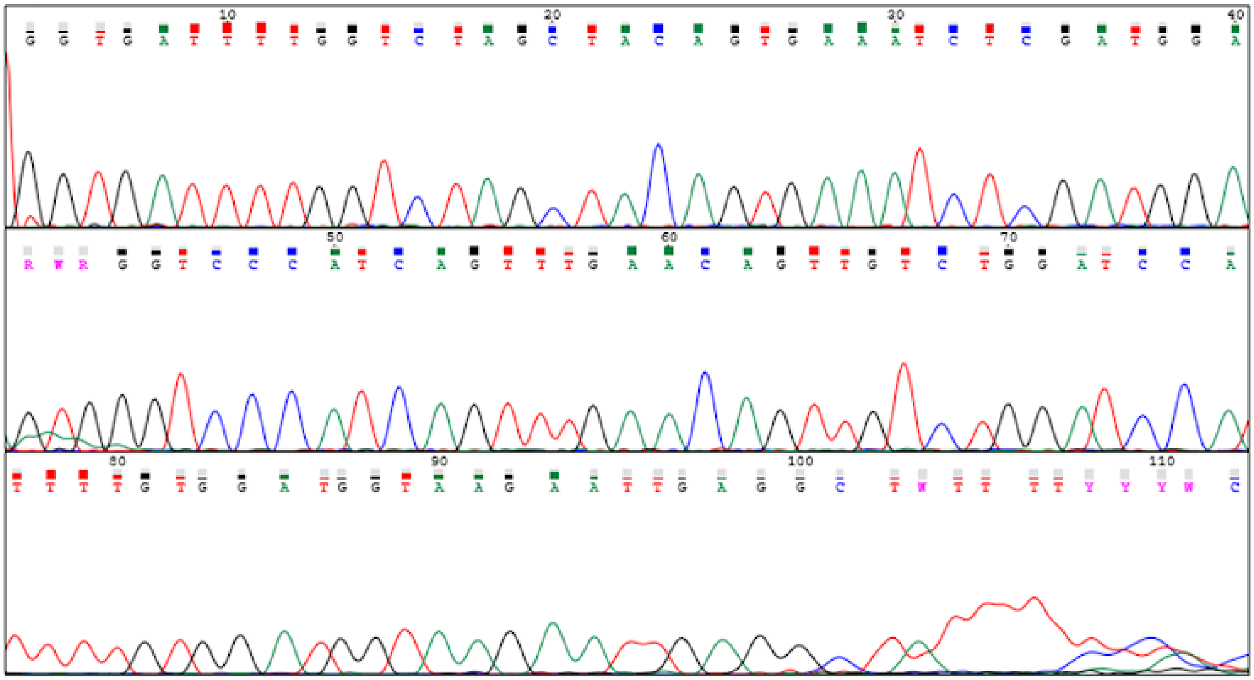

QUESTION 5

7. MPSS01 B5 — 2 points

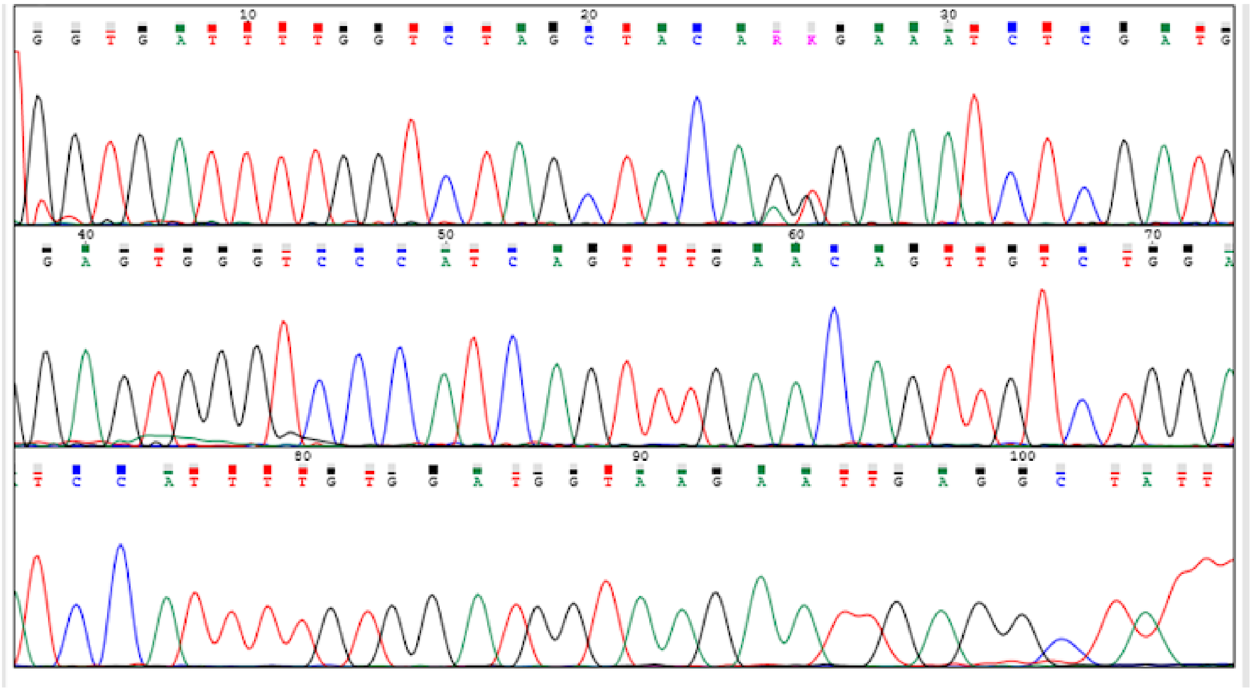

This content is neither created nor endorsed by Google.

Google Forms
