# Supplementary material for: Impact of COVID-19 on quality checks of solid tumor molecular diagnostic testing-A surveillance by EQAS provider in India
Source: PLoS One. 2022 Sep 22;17(9):e0274089. doi: 10.1371/journal.pone.0274089 (PMC9498956; doi:10.1371/journal.pone.0274089)
Supplement: S1 Table — (DOCX) [file pone.0274089.s005.docx]

S3 Table: Calendar schedule of MPQAP EQAS for the year 2020-2021

| **Month** | **Module** | **Test** |
| --- | --- | --- |
| Apr-2020 | FISH | *Her2/neu* |
| May-2020 | Gene Sequencing | *BRAF & RAS* |
| Jun-2020 | FISH | *EWSR1* |
| Jul-2020 | FISH | *ROS1* |
| Aug-2020 | Real Time PCR | *EGFR* |
| Sep-2020 | Gene Sequencing | *IDH1/2* |
| Oct-2020 | Gene Sequencing | *BRAF & RAS* |
| Nov-2020 | FISH | *Her2/neu* |
| Dec-2020 | Gene Sequencing | *KI T& PDGFRA* |
| Jan-2021 | Real Time PCR | *EGFR* |
| Feb-2021 | FISH | *1p19q &MYCN* |
